# Effects of Salmon-Derived Nutrients and Habitat Characteristics on Population Densities of Stream-Resident Sculpins

**DOI:** 10.1371/journal.pone.0116090

**Published:** 2015-06-01

**Authors:** Noel R. Swain, John D. Reynolds

**Affiliations:** Earth to Ocean Research Group, Department of Biological Sciences, Simon Fraser University, 8888 University Drive, Burnaby, British Columbia, Canada; Northwest Fisheries Science Center, NOAA Fisheries, UNITED STATES

## Abstract

Movement of nutrients across ecosystem boundaries can have important effects on food webs and population dynamics. An example from the North Pacific Rim is the connection between productive marine ecosystems and freshwaters driven by annual spawning migrations of Pacific salmon (*Oncorhynchus* spp). While a growing body of research has highlighted the importance of both pulsed nutrient subsidies and disturbance by spawning salmon, their effects on population densities of vertebrate consumers have rarely been tested, especially across streams spanning a wide range of natural variation in salmon densities and habitat characteristics. We studied resident freshwater prickly (*Cottus asper*), and coastrange sculpins (*C*. *aleuticus*) in coastal salmon spawning streams to test whether their population densities are affected by spawning densities of pink and chum salmon (*O*. *gorbuscha* and *O*. *keta*), as well as habitat characteristics. Coastrange sculpins occurred in the highest densities in streams with high densities of spawning pink and chum salmon. They also were more dense in streams with high pH, large watersheds, less area covered by pools, and lower gradients. In contrast, prickly sculpin densities were higher in streams with more large wood and pools, and less canopy cover, but their densities were not correlated with salmon. These results for coastrange sculpins provide evidence of a numerical population response by freshwater fish to increased availability of salmon subsidies in streams. These results demonstrate complex and context-dependent relationships between spawning Pacific salmon and coastal ecosystems and can inform an ecosystem-based approach to their management and conservation.

## Introduction

Ecosystems are connected by movements of energy and nutrients across their boundaries. Spatial subsidies can have far-reaching influences on the structure and function of recipient ecosystems, as demonstrated in a wide variety of habitats [1, 2]. Examples include the passive transport of nutrients from marine to terrestrial environments affecting terrestrial primary productivity and consumer populations (e.g., [3, 4]) as well as the influence of terrestrial inputs of detritus to streams on population dynamics of both direct consumers and their predators (e.g., [5]). Organisms can also actively transport energy across ecosystem boundaries. This has been demonstrated in freshwater-terrestrial linkages where terrestrial riparian invertebrates may enhance diets and abundance of fish in streams, while emerging aquatic insects can in turn determine distributions of riparian predators [6, 7, 8, 9].

Across the North Pacific Rim, highly productive marine ecosystems may subsidize freshwaters through the upstream movement of nutrients by annual spawning migrations of Pacific salmon (*Oncorhynchus* spp.). Semelparous Pacific salmon accumulate up to 99% of their body mass at sea before returning to freshwater systems where they spawn and die, often in high densities, delivering marine-derived nutrients to these typically nutrient-limited systems through their eggs, excretions and decomposing carcasses [10, 11, 12]. This provides strong pulsed nutrient subsidies that can affect organisms consuming salmon eggs and carcasses directly (e.g., [13, 14], but see [15]), and also influence food webs through bottom-up effects of increased primary productivity (e.g., [16, 17]), or when consumers switch their diets to salmon (e.g., [14, 18]). Counter to these effects, however, is the observation that salmon can also cause nutrient export from streams through spawning activities (e.g., [19]). Salmon disturb streambeds during nest digging, often in high densities, suspending nutrient-laden sediments in the water column that are then transported downstream [20]. Salmon smolts also export nutrients sequestered during freshwater rearing during their outmigration to the ocean (e.g., [21, 22]). Therefore, the initial slant in the literature towards salmon as net importers of nutrients to streams and riparian habitats has been tempered by evidence of nutrient export in some contexts [23, 24]. Indeed a recent meta-analysis of the effects of salmon on stream ecosystems by Janetski et al. [23] found that the high variability in responses to salmon were largely influenced by the environmental and methodological context of individual studies. In particular, variation in observed salmon effects are commonly explained by salmon biomass, stream habitats, and whether studies used carcass experiments or natural spawning runs of salmon. These authors highlighted the need to place findings within the context of individual studies and for further research investigating the effects of salmon over natural gradients in spawning salmon densities and stream habitats, as well as research on population level effects on stream-resident fish species.

Researchers have used stomach content and stable isotope analyses to trace nutrients from salmon through multiple trophic levels in streams, including periphyton, benthic macroinvertebrates, juvenile salmonids, and resident freshwater fish (e.g., [17, 18, 25, 26, 27]). These studies have often demonstrated increasing dietary contributions of salmon-derived nutrients with increasing availability of these resources (e.g., [14, 16, 17, 18]). For example, we have found that salmon-derived nutrients in sculpin diets are highest in streams with high salmon densities, through both direct consumption of eggs by adult sculpins, and via consumption of benthic invertebrates, which also have elevated salmon nutrient signatures [18]. Increased availability of salmon subsidies has also been linked to condition and growth in juvenile salmonids [14, 28, 29], and more recently, to their distribution and aggregation within freshwater habitats [30]. Bentley et al. [31] found that diet and growth of trout and grayling shifted in response to wide temporal variation in spawning salmon densities, and that these responses were mediated by in situ stream productivity. However, the effects of salmon vary widely among these studies and most relevant research has been limited in spatial scale and resolution, with largely binary results comparing consumer populations before and after, or with and without spawning salmon, often through experimental addition or exclusion of salmon carcasses [28, 32]. Such experiments need to be complemented by observations under natural conditions and across wider ranges in naturally spawning salmon densities (e.g., [23, 33]).

Aside from food availability, aspects of physical habitat and water chemistry such as temperature and pH are among the most important factors influencing freshwater fish populations (e.g., [34, 35]), and may further mediate the influence of salmon nutrient subsidies. Indeed, ecosystem productivity, habitat size, and physical habitat heterogeneity have all been found to be important [16, 36, 37, 38, 39]. Despite this, no study to date has tested for population level effects of salmon subsidies on fish in recipient ecosystems across a wide range of spawning salmon densities, and little is known about how these effects might be mediated by habitat characteristics [23]. The few studies that do exist have focused largely on responses by juvenile salmonids (e.g., [13, 15]). However, other resident fish such as freshwater sculpins (*Cottus* spp.) often comprise the majority of density and biomass in freshwater fish assemblages and may be influenced by both direct and indirect salmon nutrient subsidies throughout their lives, over multiple years. Consequently, sculpin populations are likely to respond strongly to salmon nutrient availability [40, 41].

In this study we surveyed populations of resident freshwater coastrange (*Cottus aleuticus*), and prickly (*C*. *asper*) sculpin in coastal salmon spawning streams. Our aims were to test the relative effects of natural gradients across streams in spawning pink (*Oncorhynchus gorbuscha*) and chum (*O*. *keta*) salmon densities, physical habitat, and water pH, on the biomass and population densities of these species, and to determine whether habitat mediated the effect of salmon (hypotheses in Table 1). We hypothesized that increasing availability of salmon-derived nutrients to streams, which contributes to increased food resources, both directly (eggs and alevin) and indirectly (increased benthic invertebrate production) [18], would translate into higher population densities of sculpins in streams with higher spawning salmon densities. We further hypothesized that variation in physical habitat characteristics such as stream substrates, gradient, and large wood density, as well as canopy cover would influence population densities of both species, and potentially mediate the effect of salmon spawning densities, depending on habitat associations of each sculpin species. For example, prickly sculpin are typically associated with pools and physical cover, and coastrange sculpins associated with shallower, faster areas of streams such as riffles and runs [42, 43]. Also low water pH can be toxic to freshwater fish with negative effects such as increased mortality, and decreased growth and reproduction leading to lower population levels at levels below 6 (e.g. [44, 45, 46, 47]). As we observed a wide range in pH in our study streams (4.8–6.4), we also predicted that sculpin densities would be higher in streams with high water pH.

**Table 1:** Predictions for the potential influence of: 1) salmon metrics 2) environmental variables on sculpin numerical and biomass densities.

Variable	Mechanism	Direction	Metric	References
**1)** Most recent salmon spawning density	Most recent salmon nutrient input provide direct and indirect resource subsidy to resident fish which may increase their population densities	Positive	Most recent autumn pink + chum adult salmon spawning density (kg m^-2^)	[17]
5 yr mean salmon spawning density	Indicates overall and legacy effect of salmon nutrient inputs to ecosystem over long term and effect of salmon subsidies on resident fish populations over time	Positive	Mean 2006–2010 autumn pink + chum adult salmon spawning density (kg m^-2^)	[17]
**2)** Watershed size	Positively associated with primary productivity, terrestrial input, foraging area, leading to increased resource availability but negatively associated with densities of some stream fish and therefore may weaken influence of salmon	Negative	PCA of mainstem and tributary length, bankfull width and catchment area	[73–75]
Substrate	Size ranges associated with inter-substrate movement and foraging by sculpin; related to habitat for sculpin and whether they have direct access to salmon eggs/alevins, which may mediate the strength of salmon effects	Positive	PCA of sculpin foraging substrate (% coarse gravel + % small cobble + % large cobble); mean, and SD for substrate size	[51, 55, 68, 76]
Pools	Number and area of pools related to habitat heterogeneity, primary productivity and consumer foraging, positively associated with prickly, negative with coastrange sculpin habitats	Positive for prickly and negative for coastrange sculpins	Pools per 100m; % pool area	[73, 77]
Undercut bank	Undercut banks associated with prickly sculpin habitat and stream fish habitat in general	Positive for prickly and negative for coastrange sculpins	% undercut banks	[73, 74]
Gradient	Higher gradient channels tend to have lower productivity and are often negatively associated with sculpin habitat, and they may also flush out nutrients more quickly	Negative	Mean gradient degrees; % high gradient habitat	[34, 50, 68]
Large wood density	Associated with channel heterogeneity and sculpin foraging habitat, positively associated with stream fish densities through providing habitat and cover	Positive	Pieces of large wood pieces per 100 m	[35, 78, 79]
Canopy cover	Negatively associated with primary productivity and subsequently to stream fish population densities—proxy for light availability and thus stream productivity, may limit salmon effects on stream productivity	Negative	Canopy cover	[34, 67, 68]
pH	Low stream water pH is toxic to fish and has been shown to negatively affect stream fish populations densities through increased mortality and decreased growth and reproduction	Positive	2006–2009 mean autumn pH	[46, 47]

## Material and Methods

### Ethics Statement

All counts of spawning chum and pink salmon, and capture, collection, and handling of sculpins were approved and conducted in compliance with the guidelines and policies of the Canadian Council on Animal Care (Simon Fraser University approval number 1014B-07).

### Study sites

**Fig 1:**
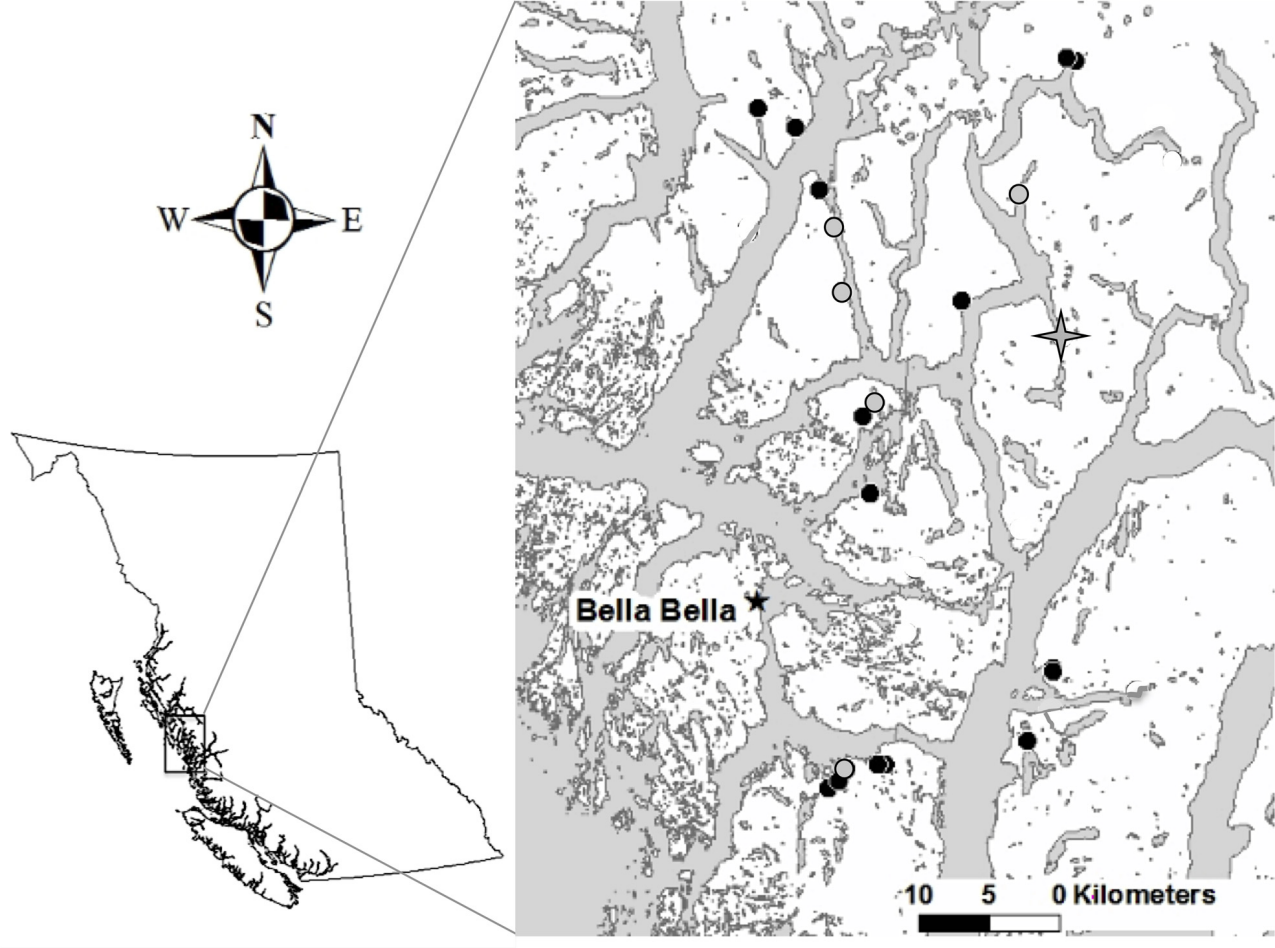
Locations of the 21 streams in the Great Bear Rainforest region of coastal British Columbia, Canada. Streams surveyed in 2010 are shown with black circles, those surveyed in 2010 and 2011 are shown with grey circles, and the stream surveyed only in 2011 is shown with a grey star.

We studied 21 streams on coastal islands and mainland inlets in the Great Bear Rainforest region of British Columbia’s central coast (Fig. 1, S3 Table). These streams were selected based on availability of data from previous research and habitat surveys, and to include systems spanning several natural gradients: 1) a wide range of spawning salmon abundance, from zero to more than 60,000 combined pink and chum salmon returning to streams in a given year; 2) watershed size, and 3) habitat structure and heterogeneity (e.g., substrate composition, gradient, density of large wood). These streams are in coastal, conifer-dominated temperate rainforest in the Coastal Western Hemlock Biogeoclimatic Zone, and are characterized by relatively few anthropogenic impacts aside from some selective logging in the 1940s [38]. Surveyed stream areas were located within lower reaches of salmon spawning streams, and three streams where no spawning salmon have been observed, including one site (Ripley Bay) located above an impassable waterfall at the mouth of the stream. This site had landlocked populations of both sculpin species which have been documented elsewhere (e.g., [43]).

### Sculpin population surveys

We surveyed sculpin populations during low-flow conditions in 20 streams during July and August 2010, five of which were re-surveyed along with one new stream in August 2011. In each stream we surveyed a single lower reach within the length of stream in which salmon spawn. Reaches were well above tidal influence and included representative habitat types of both sculpin species (coastrange sculpins—riffles and runs, prickly sculpins—pools and undercut banks). Where possible, reach lengths were five times the average wetted width of each stream (distance between water’s edge on both sides of the channel); in exceptionally large and small streams, surveyed area relative to wetted width was decreased or increased, respectively, to maintain a manageable survey area and to include representative reaches of streams. The average area of surveyed reaches was 139 m^2^ (range, 45–624 m^2^), calculated as the average reach length × average wetted width, minus any areas not covered by water. Surveyed reaches were blocked with 5-mm mesh barrier nets at the upper and lower boundaries, and three to five removal passes [48] were made with a backpack electrofishing unit (LR-24 and model 12-B, Smith-Root, Vancouver, WA, USA), supported by two people with dip nets. Exceptions were two sites where only one removal pass was conducted and one site where only two passes were possible due to time and equipment constraints. Nonlethal sampling of all fish entailed species identification and measurements of standard length (mm) and wet weight (to the closest 0.1 g). Fish were released back into surveyed reaches after electrofishing surveys were completed.

We estimated population sizes within survey areas using a hierarchical Bayesian model similar to that described by Wyatt [49]. This model assumes that the site-specific parameters (catchability and population abundance) are random variables that come from hyper-distributions at the regional level. This approach generally improves precision of estimates for multiple-pass sites and allows estimates to be obtained at single- and two-pass sites. We could not use this hierarchical approach for prickly sculpins due to sparse data (small catches and poor depletions at most sites), so population estimates for this species were calculated using maximum likelihood multiple-pass depletion methods developed by Carle and Strub [48]. Estimates obtained with the two methods were compared for coastrange sculpins and they proved to be very similar (S1 and S2 Figs.). Although coastrange sculpins were found in all 21 of the surveyed streams and prickly sculpins were found in 14 streams, it was only possible to calculate population estimates for 20 and 12 streams, respectively, due to low abundance or poor depletions. A lack of detection did not necessarily mean a species was not present in a given stream, as in several systems, prickly sculpins were not found during electrofishing surveys, despite having been caught using g-traps the previous autumn. Therefore, only streams with population estimates were included in subsequent analyses for each species. Sculpin densities were then calculated by dividing these population estimates by reach area to give fish m^-2^. We also calculated sculpin biomass (g m^-2^) by multiplying population estimates by the mean masses of each species divided by reach area. Although juveniles of these sculpin species typically rear in estuaries, adults are more sedentary and closely associated with freshwater habitats [43, 50]. As our streams are small, coastal systems typically separated by considerable distances of marine environment (~0.2–65 km), there was unlikely to have been any significant movement by sculpins among them. Therefore, we considered populations and the effects of spawning salmon upon them to be discrete among streams. Although other studies have shown aggregative responses by freshwater fish, including sculpins, to areas where salmon spawn (e.g., [30, 51]), here, we consider that these sculpin population estimates are more likely to indicate overall population densities of the stream rather than simply seasonal aggregations for the following reasons: 1) Sculpin population surveys were conducted in the summer (July—August) over a month after peak alevin emergence (April—May) and one to two months prior to peak pink and chum spawning (late September—October) observed in this region [18, 40]; 2) other studies have shown that densities of both species are higher in downstream reaches, likely due to estuary rearing of sculpins typical in coastal streams [43]; 3) roughly 40% of the sculpins caught in our surveys were below the body size threshold associated with direct consumption of salmon eggs or alevin observed in other studies (e.g., [40, 51]) (S5 Fig.).

### Salmon population data

From 2006–2010, autumn salmon enumeration was conducted jointly by people from Simon Fraser University, the Heiltsuk First Nation, and Fisheries and Oceans Canada from late August to early November. Spawning salmon counts were done by stream surveys on foot, with the aim of visiting each stream at least three times during the spawning season. Abundance estimates were calculated using area under the curve or peak live + dead abundance, which were highly correlated estimates with comparable means [38, 52]. In the few cases where pink or chum abundance estimates for a stream were missing for a given year (n = 5), we used predicted values from hierarchical linear regressions of abundance estimates based on regional trends from 2006–2010. This allowed us to include two streams missing 2009 salmon estimates, and 3 streams missing salmon data in a previous year (2006–2008) needed to calculate multi-year metrics (see below). We calculated biomass for pink and chum salmon for each year as the estimated number of adults returning to a stream multiplied by regional adult mean mass calculated from measurements taken during annual surveys (kg). To account for the potential effect of spawner density, we calculated this metric as kg m^-2^ by dividing adult salmon biomass by salmon spawning area, measured as the distance from the mouth of the stream to the furthest point upstream where spawning salmon were observed × average wetted width of the stream. We considered both salmon densities during the year prior to stream surveys (e.g., 2009 for 2010 sculpin surveys), to characterize salmon nutrient contribution from most recent spawning events, and as 5-year means (2006–2010), which dampens out annual variation and reflects a more general and temporally robust characterization of salmon nutrient contributions to each stream. We also included the sum of salmon density estimates over the two spawning seasons prior to sampling (2008–2009 or 2009–2010), with the contribution of estimates from two years prior down-weighted by a negative exponential function describing the rate of salmon-derived nutrient loss from the watershed [16]. This was done for rates of loss that corresponded to salmon nutrient half-lives in a watershed of six, 12, and 24 months. All salmon metrics were highly collinear and because previous autumn salmon density performed better than multi-year metrics (S1 Table), and results did not differ among these metrics, we present results for only previous autumn salmon density. Although present in several of these systems, adult steelhead and coho salmon were not included in analyses due to their extremely low abundance in the reaches of the streams where we worked.

### Stream habitat characterization

Based on previous research, we hypothesized that variation in stream habitats such as watershed size, habitat structure (e.g., substrate, large wood density, and presence of pools and undercut banks), light availability, stream gradient, and water pH are likely to affect sculpin population biomass and densities, contingent on habitat associations and tolerances of each species, and overall stream productivity (Table 1). We also hypothesized that such habitat variation could mediate observed effects of salmon by influencing the availability of their nutrients to sculpins. Most habitat metrics were measured during stream habitat surveys carried out in 2007, with additional streams and missing metrics added through surveys in 2011. Habitat surveys were conducted for a reach of stream, the length of which was roughly 30 times the average wetted width, starting just above the estuary margin. A reach was divided into four equal sections from which three transects were selected randomly, to a total of 12 transects at which measurements were taken. In all sites, reaches where habitat surveys and salmon counts occurred had strong spatial overlap, with sculpin surveys conducted within lower to mid sections of the larger survey reaches. These surveys covered a considerable portion of the suitable habitat for both sculpins and salmon, as most streams were relatively short, steep gradient systems where salmon spawning and sculpin habitat typically occurred in lower reaches (e.g., < 1 km in length), below barriers to upstream migration. To characterize watershed sizes, stream length and catchment area were determined from area maps and iMapBC (http://www.webmaps.gov.bc.ca), and bankfull width was measured during habitat surveys at each transect and averaged for the entire habitat reach. Stream habitat structure metrics included large wood pieces per 100 m of channel within the reach (large wood density), % of the habitat reach consisting of high gradient habitat (falls, riffles, runs, cascades, step pools, and step runs), stream gradient (measured with a clinometer, and averaged over all transects in each stream), % of transects where undercut banks were present (% undercut bank), pools per 100 m of channel within the reach, % of the reach area made up of pools (% pool area) and substrate composition. Substrate was measured at each transect and categorized using Wolman pebble counts [53]. Canopy cover was measured with a densiometer, and averaged over the 12 transects in each stream. Water pH was measured biannually during summer and autumn between 2006 and 2011 using a handheld meter. Measurements were taken in areas of running water in the lower, mid, and upper sections of habitat reaches and averaged across all years.

We reduced our set of explanatory variables using methods described by Zuur et al. [54]. We examined multicollinearity among habitat variables using variance inflation factor (VIF) (S2 Table). VIF quantifies multicollinearity through ordinary least squares regression analysis that measures the level to which the variance of an estimated regression coefficient is increased due to collinearity among explanatory variables. Highly collinear variables (VIF > 4) were either combined with similar variables through principal component analysis (PCA) or dropped from subsequent analyses.

We used the first principal component of stream length, catchment area, and bankfull width as a metric of watershed size [38, 52]. This axis explained 94% of the variation in these variables, and all three variables loaded positively with eigenvalues greater than 0.5. We characterized stream habitat structure through a number of metrics described above: large wood density within the reach, % of the habitat reach consisting of high gradient habitat, % undercut bank, pools per 100 m of channel within the reach, % pool area, and substrate. We further characterized foraging substrate as the % substrate in size classes of “coarse gravel”, “small cobble”, and “large cobble”, corresponding to the size range associated with inter-substrate foraging by sculpins on salmon eggs, alevins, and benthic invertebrates [51, 55]. Following methods by Bain et al. [56], substrate types were also coded in order of coarseness, with the mean and SD of coded values within each stream expressing substrate size and heterogeneity, respectively. Because these substrate variables were highly correlated with one another, they were also combined through their first principal component, which explained 80% of their variance, and on which foraging substrate and coded means loaded negatively (< -0.5), and coded SD loaded positively (0.63). We also used canopy cover as a proxy for light availability, and included stream gradient as a physical habitat variable. Due to sparse data, only mean 2007–2009 autumn water pH levels were available for all streams in this study, which were highly correlated with 2010 pH in streams where the data were available. We also considered water temperature in our analyses as it may have strong influences on freshwater fish populations and their trophic interactions with spawning salmon (e.g., [39]). However, adequate water temperature data was available for less than half of our surveyed streams and did not meaningfully influence our results, so it was dropped from our final analyses.

### Data analysis

We used multiple linear regression and model selection via Akaike information criterion corrected for small sample sizes (AICc) to evaluate the relative effects of salmon density and other explanatory variables on sculpin population densities in the summer of 2010 (coastrange sculpins n = 19 streams; prickly sculpins n = 11 streams). The following explanatory variables were included based on *a priori* hypotheses (Table 1): previous autumn combined pink and chum salmon spawning density (kg m^-2^); watershed size (PC1 scores); substrate (PC1 scores); large wood pieces per 100 m; pools per 100 m; % pool area; % high gradient habitat; % undercut bank; canopy cover; mean gradient degrees; and mean autumn water pH; as well as interactions between salmon density × habitat variables. Salmon and sculpin densities were transformed (log[variable + 0.1]) to meet model assumptions of normality.

We evaluated the relative support for these hypotheses using an all-submodels combination approach (n = 242), restricting our candidate model set to those models having all combinations of only pre-hypothesized interactions and a maximum of three parameters (including interactions) [57, 58]. In this approach, model uncertainty and the relative effects of explanatory variables are assessed using AICc and multi-model averaging (e.g., [59, 60]). AICc assesses the relative descriptive power of a model containing different combinations of variables based on the principle of parsimony by balancing optimal fit with the number of parameters used, where the lowest AICc score corresponds to the top performing model [60]. Where a number of high-ranking models are weighted similarly, indicating high uncertainty in model performance, and relatively little support for individual models, model averaging can be used to incorporate model uncertainty into estimated parameters [59]. We used the MuMIn package in R [61] to compete models against one another based on Δ AICc and AICc weights (*w*_*i*_), and to calculate multi-model averaged parameter estimates from the 95% confidence set of candidate models (e.g., [58, 59]). We also calculated relative variable importance as the sum of AICc weights from all models containing the variable of interest in this 95% confidence set. To compare among all parameters and interpret the main effects in conjunction with interaction terms, we conducted the above analyses after standardizing continuous explanatory variables by subtracting global means from each value (centering) and dividing by two times the SD (scaling) [62]. We also conducted these analyses without our highest salmon density stream in order to assess its influence on our results. As this did not meaningfully change our results and as log transforming salmon density data lowered its influence, we only present results from analyses including this site.

Finally, we tested how temporally robust the best performing salmon + habitat models were by predicting 2011 sculpin densities from data collected in previous years. For this analysis we used non-standardized continuous explanatory variables to maintain the predictive utility of models. We used 2010 coastrange sculpin densities and biomass, 2009 salmon spawning densities, and habitat variables (n = 19; top habitat + salmon models, Table 2) to apply model parameter estimates to 2010 salmon densities to predict sculpin densities and biomass in 2011. We then compared our observed and predicted estimates (both log transformed and back transformed values) for the subset of six streams surveyed in 2011 through linear regressions (S3 Fig.). All statistical analyses were conducted in R [63] except for hierarchical Bayesian depletion estimates, which were conducted in WinBUGs [64].

**Table 2:** Results for model selection using AICc showing high-ranking linear regression models (Δ AICc < 3) for coastrange and prickly sculpin biomass and densities.

Response	Model	K	Δ AICc	W_i_	R^2^
**Coastrange sculpin density**	pH + % pool area + gradient	5	0	0.2	0.67
	pH + gradient	4	2.42	0.06	0.55
	salmon + % pool area	4	2.68	0.05	0.54
	salmon + pH + % pool area	5	2.99	0.05	0.62
					
**Coastrange sculpin biomass**	salmon + pH + % pool area	5	0	0.1	0.68
	pH + % pool area + % high gradient habitat	5	0.97	0.06	0.67
	pH + % pool area + gradient	5	1.11	0.06	0.67
	salmon + % pool area + canopy cover	5	1.16	0.06	0.66
	salmon + % pool area	4	1.32	0.05	0.59
	salmon + pH + large wood density	5	2.47	0.03	0.64
	pH + large wood density + canopy cover	5	2.63	0.03	0.64
	salmon + % undercut bank + watershed size	5	2.72	0.03	0.64
	salmon + watershed size	4	2.73	0.03	0.56
	salmon + % pool area + watershed size	5	2.78	0.03	0.63
	pH + large wood density	4	2.86	0.02	0.55
					
**Prickly sculpin density**	large wood density + canopy cover	4	0	0.19	0.78
	large wood density + canopy cover + pools per 100 m	5	1.01	0.11	0.88
	large wood density + % pool area	4	1.27	0.1	0.75
	large wood density + canopy cover + % high gradient habitat	5	1.74	0.08	0.87
	large wood density + % pool area + substrate	5	1.96	0.07	0.86
	large wood density + canopy cover + % undercut bank	5	2.49	0.05	0.86
	large wood density + canopy cover + % pool area	5	2.5	0.05	0.86
					
**Prickly sculpin biomass**	large wood density + canopy cover	4	0	0.09	0.59
	null model	2	0.55	0.07	0
	large wood density	3	0.65	0.07	0.29
	large wood density + pH + substrate	5	0.66	0.07	0.77
	% high gradient habitat	3	1.62	0.04	0.23
	substrate + watershed size	4	1.98	0.03	0.51
	watershed size	3	2.21	0.03	0.19
	% pool area	3	2.32	0.03	0.18
	canopy cover	3	2.51	0.03	0.16
	pools per 100 m	3	2.55	0.03	0.16
	gradient	3	2.59	0.03	0.16
	substrate	3	2.69	0.02	0.15
	large wood density + pH	4	2.81	0.02	0.47
	% high gradient habitat + watershed size	4	2.94	0.02	0.46

Candidate sets contained all combinations of parameters: single-year salmon spawning density (salmon), autumn pH, watershed size, canopy cover, and physical habitat metrics (see Table 1), and were restricted to including only interactions between salmon density and covariates, and up to 3 stream level parameters including interactions.

## Results

**Fig 2:**
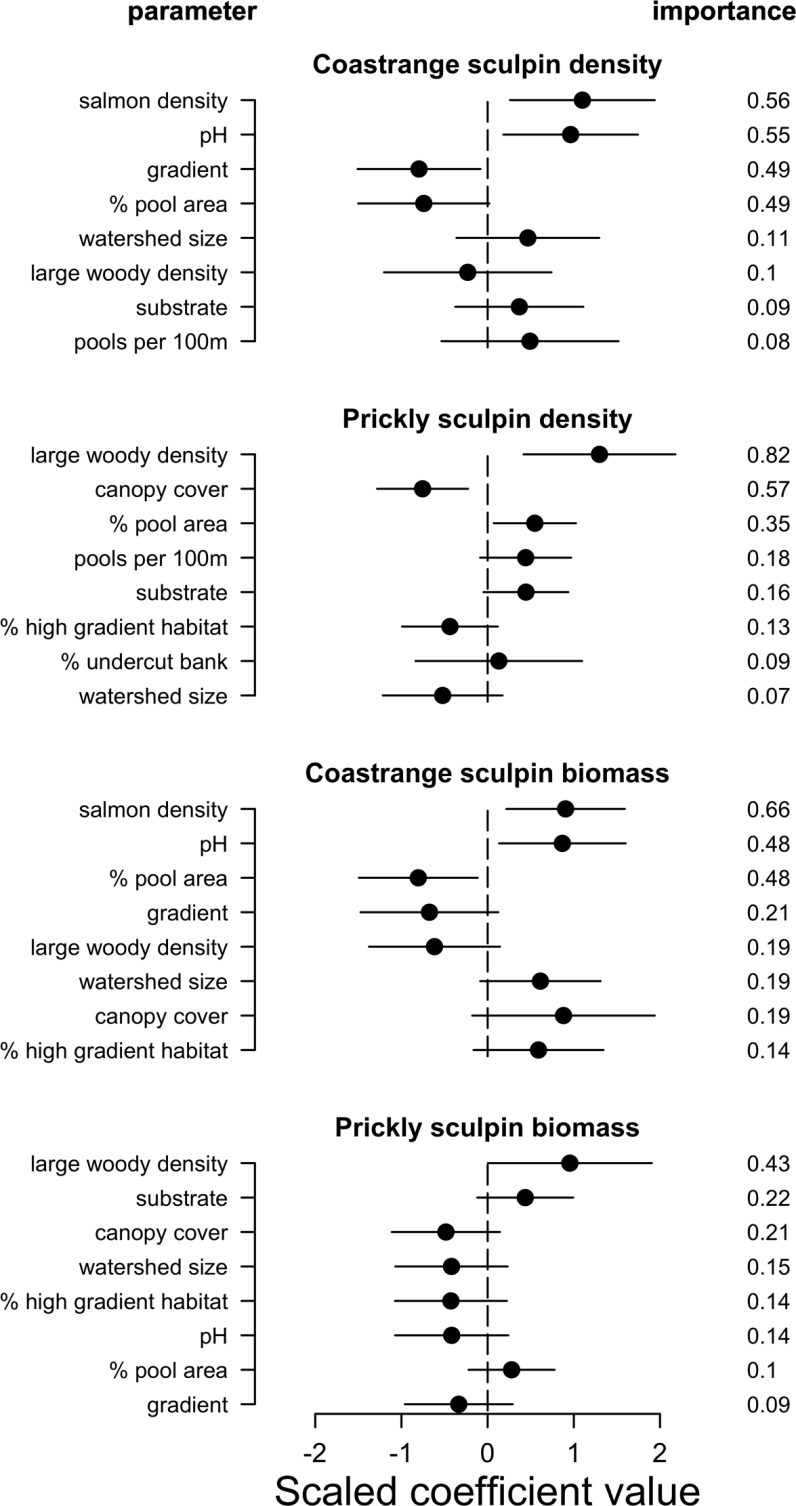
Scaled parameter estimates (circles) with 95% unconditional confidence intervals (lines) from the 95% confidence set of averaged multiple linear regression models of the effect of pink and chum salmon spawning density and stream habitat on coastrange and prickly sculpin densities (n = 81 and 60 respectively) and biomass (n = 85 and 87 respectively). Parameters are ordered by their relative importance (indicated on right) to the averaged model on a scale of zero to one with only the top eight parameters for each response variable indicated. Results for all parameters are included in S4 Fig.

Coastrange sculpins (*C*. *aleuticus*) were caught in more streams than prickly sculpins (*C*. *asper*) (21 [100%] versus 14 [67%] streams, respectively), and were much more abundant in the reaches that we surveyed (S3 Table). Based on personal observations in the field and length-class frequencies, and consistent with previous studies (see [43]), adult prickly sculpins were generally larger than coastrange sculpins (73.2 ± 22.3 versus 51.6 ± 17.9 mm standard length respectively). The relative effects of salmon spawning density, pH, and physical habitat differed between species as well as between density and biomass estimates (Fig. 2, S4 Fig.).

There were numerous high-ranking models (Δ AIC < 3; Grueber et al. 2011) describing sculpin densities and biomass (Table 2). Aside from the top-ranking model for coastrange sculpin density which contained pH, % pool area, and gradient, low *w*_*i*_ and similar Δ AICc values indicate high uncertainty and relatively little support for any one model. Interactions between salmon and habitat variables were also absent from all high-ranking models aside from two models describing coastrange sculpins biomass, which included either an interaction between salmon and % pool area or canopy cover (Table 2). These top models generally explained a considerable amount of the variation in sculpin densities and biomass among streams, particularly those describing prickly sculpin densities (R^2^ = 0.75–0.88), while biomass models for this species were highly variable with R^2^ values between 0 and 0.78.

Salmon density, pH, % pool area, and gradient were the most common variables in high-ranking models describing coastrange sculpin densities and biomass, though salmon density was not included in the top two density models for this species. Stream gradient and substrate were also present in at least one high-ranking model for coastrange sculpin density, while gradient, canopy cover, large wood density, watershed size, pools per 100 m, % undercut bank, and % high gradient habitat were included in high-ranking models describing biomass of this species. In contrast, large wood density was the most common variable in models describing prickly sculpin density and biomass, followed by canopy cover and pool habitat variables (Table 2).

**Fig 3:**
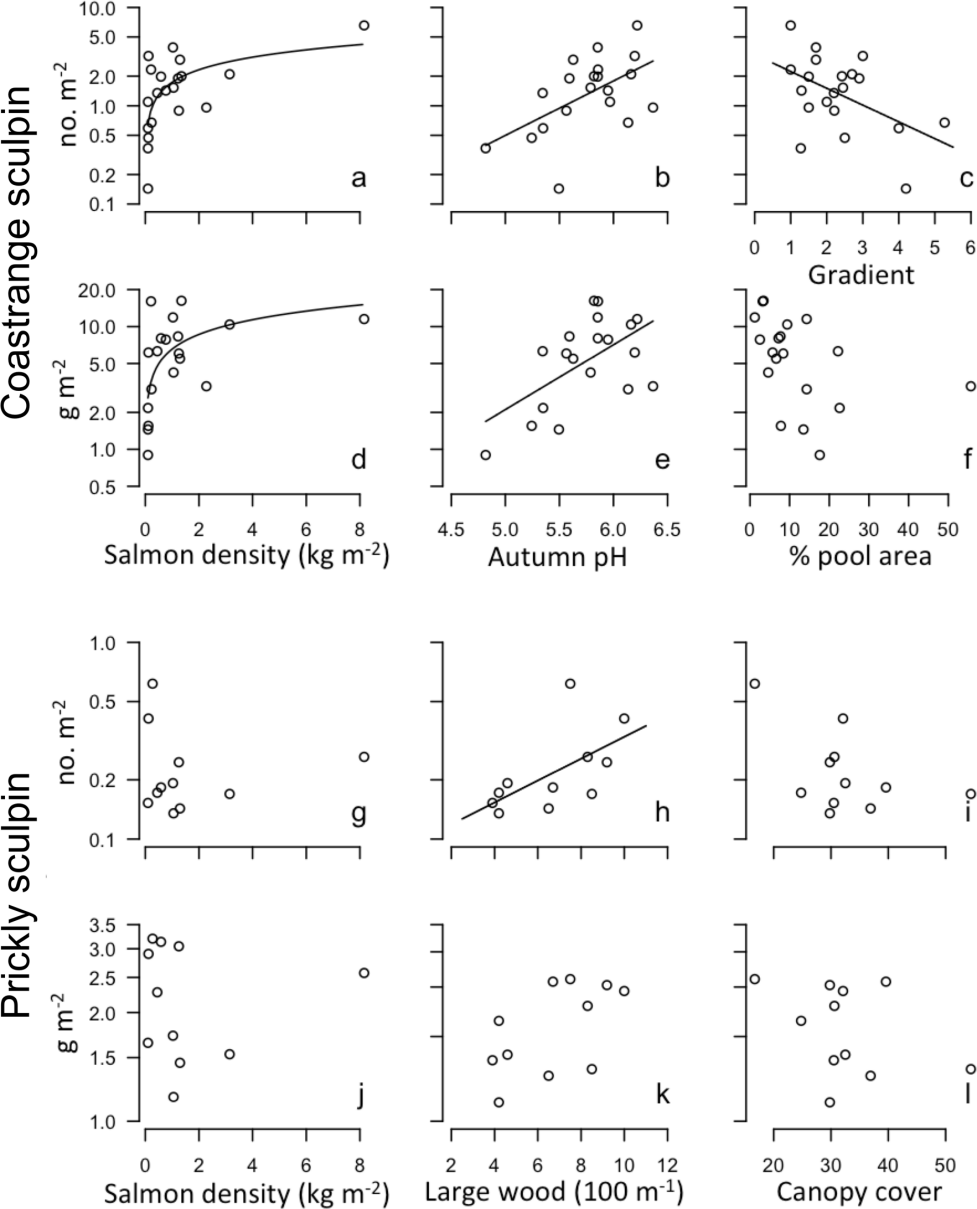
Row 1: Bivariate plots of coastrange sculpin density (log[no. m-2]) versus: a) 2009 salmon density (kg m-2), b) 2006–2009 mean autumn water pH, c) mean gradient degrees. **Row 2: coastrange sculpin biomass (log[g m-2]) versus d) 2009 salmon density, e) 2006–2009 mean autumn water pH, f) % pool area. Row 3: Bivariate plots of prickly sculpin density (no. m-2) versus g) 2009 salmon density, h) large woody debris pieces 100 m-1, i) canopy cover. Row 4: bivariate plots of prickly sculpin biomass (g m-2) versus j) 2009 salmon density, k) large wood pieces 100 m-1, l) canopy cover.** Regression lines are included in plots where significant relationships were observed (p <0.05). These lines are curved for plots with salmon density as this variable was back transformed to demonstrate the non-linear nature of these relationships.

As predicted, salmon density and water pH had strong positive correlations with coastrange sculpin densities and biomass in streams, explaining variation among streams better than any other variables (relative importance = 0.48–0.66). In post-hoc analyses when we dropped pH from competing models, the importance of salmon density also increased considerably (relative importance > 0.9), and was present in all top-ranking models, with the only consistently positive model averaged coefficients. Coastrange sculpins were also more abundant in low gradient streams with few pools and less large wood (Figs. 2, 3; Table 2). In line with our predictions, prickly sculpin biomass and densities were higher in streams with more large wood, more pools, less canopy cover, and weaker gradients, with large wood density and canopy cover being by far the best variables at explaining differences across streams (Figs. 2, 3). In contrast to results for coastrange sculpins and contrary to our predictions, however, neither pH nor salmon density had appreciable relationship with prickly sculpin densities (Figs. 2, 3).

Our results for coastrange sculpins were also temporally consistent, with the observed 2011 densities and biomass correlated with those predicted from top salmon + habitat model parameters derived from 2010 sculpin densities or biomass, 2009 salmon densities, and habitat data fitted with 2010 salmon density data (S3 Fig.). Intercepts and slope of observed versus fitted values were not significantly different from zero and 1, respectively suggesting low bias and high consistency in these models (e.g., [65]).

## Discussion

We found that the influence of salmon, habitat, and pH on sculpin population densities across our 20 streams surveyed in the summer of 2010 varied considerably between the two sculpin species, highlighting the importance of considering species and context-specific responses to stream habitat and salmon subsidies. In general, coastrange sculpins were found in the highest densities in streams with greater spawning densities of pink and chum salmon adults, high water pH, less area covered by pools and large wood, and lower gradients. In contrast, prickly sculpin densities were higher in streams with more large wood and pools, and less canopy cover. Our results suggest that coastrange sculpin density and biomass may benefit from increased availability of salmon nutrients in these streams. Based on a lack of interaction terms between salmon density and habitat in top-ranking models, we found little evidence for habitat mediating the relationship between spawning salmon and sculpin densities. However, a number of habitat variables played considerable independent roles in determining sculpin populations, and therefore need to be considered because of their potential to mask the effects of salmon.

Numerous studies have shown that nutrients from spawning salmon are consumed by freshwater fish (e.g., [14, 18, 25, 26]). However, few studies have provided compelling or consistent evidence that increases in salmon-derived nutrients actually affect these fish populations, and results from these studies vary widely due to the context in which they were conducted [23]. We know of seven studies that have evaluated effects of salmon subsidies on freshwater fish biomass, condition, or growth [13, 15, 18, 30, 31, 66, 67]. Of these, five documented such effects across natural variation in salmon densities, two of which focused on juvenile salmonids. Bilby et al. [13] compared two reference streams with low spawning salmon densities to two streams where naturally spawning salmon carcasses were augmented by hatchery carcasses in autumn and found that condition of juvenile salmonids increased with carcass additions when salmon were spawning. Similarly, Wipfli et al. [66] found increased growth in juvenile salmonids with carcass additions in artificial stream channels and natural stream reaches, and Denton et al. [30] found that growth rates of resident salmonids increased with the availability of salmon eggs and blowfly larvae associated with naturally spawning salmon in ponds. Bentley et al. [31] recently showed that growth rates of resident trout and grayling were positively related to natural salmon spawning densities in two streams over the course of over a decade, and that this relationship was influenced by *in situ* stream productivity. Swain et al. [18] also found some evidence that sculpin condition increased with salmon densities across over 30 coastal streams. In contrast, Wilzbach et al. [67] and Harvey and Wilzbach [15] found that density and biomass of juvenile salmonids were not noticeably affected by salmon carcass additions.

While other studies have found seasonal, aggregative responses by stream fish to spawning salmon (e.g., [30, 51]), to our knowledge, this is the first to provide evidence of differences in population abundance of stream fish with natural salmon spawning densities. Although we cannot rule out an additional role of aggregative responses by sculpins to food sources associated with spawning salmon, our sculpin surveys were conducted 1–2 months after outmigration of alevins and 1–2 months before the spawning salmon arrived, and much of the coastrange sculpin densities and biomass observed in these streams were composed of individuals too small to directly consume salmon eggs (e.g., < 50 mm in length, [51]). In a concurrent study within these systems [18], we found strong evidence that salmon subsidies are consumed both directly by sculpins (e.g., eggs) and indirectly through lower trophic levels.

We found that salmon density had a considerable positive relationship with both biomass and densities of coastrange sculpins in our survey reaches, but not those of prickly sculpins. The higher importance of salmon density over habitat in explaining biomass but not numerical densities of coastrange sculpins (based on a lack of salmon density in top, higher weighted models for the latter), suggests a stronger influence of food availability on sculpin growth and size, and stronger effect of habitat on numerical abundance, consistent with the literature (e.g., [30, 31, 68]). These differences may also reflect differences in the size structure of sculpin populations among streams. As Wilzbach et al. [67] found for juvenile salmonids, we found that habitat often played an equal to, or greater role than salmon subsidies in determining sculpin populations.

Low water pH has been shown to strongly affect freshwater fish abundance and biomass, potentially masking other effects of habitat at toxic levels below 6.0 [46, 47]. Therefore, it is not surprising that pH had one of the strongest effects on coastrange sculpin populations given its wide range in these streams (4.82–6.37). Contrary to our predictions, however, pH had a negligible effect on prickly sculpin populations. Freshwater sculpins have been reported to be highly tolerant of water pH and prickly sculpins in particular have adapted to most freshwater habitats including standing and brackish waters where pH levels can be low [43, 69].

Increased pool habitat, the presence of large wood, and open canopy cover have also been linked to greater abundance and body size of many stream fish through increased nutrient availability leading to elevated primary and secondary production (e.g., [47, 70]). We confirmed the prediction that prickly sculpins would be more abundant in streams with more pools and large wood and less canopy cover (linked to higher productivity), whereas we found the opposite effect of wood and pools for coastrange sculpins, consistent with their association with higher velocity stream habitats such as riffles and runs [42]. In line with findings by Hawkins et al. [34], we also found that stream gradient had a negative influence on both sculpin species, particularly coastrange sculpin densities, which we hypothesized would be due to decreased habitat, as well as lower productivity and salmon nutrient retention decreasing food availability in higher gradient systems (e.g., [71]).

Despite finding a strong influence of some habitat characteristics on both sculpin species, and a considerable positive effect of salmon spawning density on coastrange sculpins, we found little support for stream habitat mediating the observed effects of spawning salmon densities. Wilzbach et al. [67] also found strong independent influences of habitat on densities and biomass of juvenile salmonids, but in contrast to our results for coastrange sculpins, they did not observe an effect of salmon. They attributed this to canopy cover limiting light availability necessary for primary productivity to increase with the input of salmon nutrients. Although canopy cover had a direct negative influence on prickly sculpin densities, we did find some evidence that the positive effect of salmon on coastrange sculpin biomass was lower in streams with greater canopy cover, which could reflect this same mediating effect of light limitation in our streams.

Our results from 2010 were also relatively consistent over time, with observed estimates of coastrange sculpin densities from surveys in 2011 fitting predictions from top salmon + habitat models well. Freshwater sculpins live for multiple years, so populations are inherently correlated between years, however the relatively good fit between observed versus fitted density estimates suggests some temporal robustness of our sampling methodology and statistical results.

Although the variables we examined explained much of the variation in biomass and densities of sculpins among streams, it would be interesting for future studies to quantify food availability, including benthic invertebrates and small fishes (e.g., [68]), as well as competition with other species such as trout (e.g. [72]).

The relationships we observed between habitat variables and prickly sculpin abundance generally followed our predictions, but their lower prevalence in surveyed stream reaches and the lack of an observed effect of salmon or pH on this species may, in part, be due to our sampling methodology. Our electrofishing gear would have been more efficient in riffles that coastrange sculpins prefer than in pool and cover-oriented habitats of prickly sculpins, leading to an under representation and poor estimation of prickly sculpin populations in some streams. Anderson [73] found that, in general prickly sculpin populations are more concentrated in higher reaches of coastal streams, while coastrange sculpin densities are higher in lower reaches. That we typically surveyed lower reaches of streams may thus have created a bias towards coastrange over prickly sculpins. This, along with the highly variable results for prickly sculpin biomass (e.g., intercept-only model was the second best model for prickly sculpin biomass based on Δ AIC), gives us less confidence in the observed habitat and salmon associations for this species than for coastrange sculpins and we compare our differing results for the two species with caution. Future studies should consider alternative survey techniques and equipment such as mark-recapture methods using baited minnow traps, which are less likely to bias results for this species.

Many of our predictions for how habitat would influence sculpin biomass and density, and mediate the effect of salmon were not supported by our results. We did not find significant effects of substrate or watershed size, in contrast to studies of other freshwater fish populations (e.g., [68]) and salmon-ecosystem interactions (e.g., [38]). However, such interpretations must be placed within the context of our study (e.g., [23]); it may be that variability in these habitat characteristics among streams did not span necessary thresholds of influence, or that our metrics were too localized to detect significant effects of variables that may be important in other circumstances. Other variables such as water temperature have also been shown to strongly influence freshwater fish populations and mediate the effect of salmon subsidies on recipient consumers (e.g., [39]). Although we did not include water temperature in our study due to inadequate data and a lack of an appreciable effect in our exploratory analyses, it may be an important factor in explaining the effects of salmon on freshwater fish such as sculpin, in streams that span wider gradients in water temperatures.

Our results suggest that some freshwater fish species can be influenced by annual spawning runs of Pacific salmon (see also [22, 33]), but highlight how effects are highly context dependent, differing markedly among closely related species and potentially influenced by habitat and study methodology. We demonstrate the independent importance of habitat characteristics in determining stream fish populations and the need to consider such influences when trying to isolate the effects of salmon subsidies to recipient consumer populations. As habitat engineering and salmon carcass additions are often used in stream restoration, it is helpful to understand the impacts of salmon subsidies across a wide range of natural variation in habitats to predict the success of these programs. Furthermore, understanding how salmon subsidies impact vertebrate consumer populations in recipient food webs across natural gradients in spawning salmon returns is key to effective ecosystem-based conservation and management of wild salmon and associated freshwater systems.

## Supporting Information

S1 Fig Coastrange sculpin density estimates and 95% CI derived from maximum likelihood multiple-pass depletion methods (Carle and Strub 1978)(−o−) and estimates and 0.025 and 0.975 credible limits derived from Hierarchical Bayesian depletion models (Wyatt 2002)(—-Δ—-) for surveyed streams in 2010 and 2011. (PDF)

S2 Fig Coastrange sculpin density estimates derived from maximum likelihood multiple-pass depletion methods (Carle and Strub 1978) versus Hierarchical Bayesian depletion models (Wyatt 2002). Dotted line is the 1:1 line and solid line is the regression line between the two methods.
(PDF)

S3 Fig Observed 2011 versus predicted coastrange sculpin log(densities) (A), densities (B), log(biomass) (C), and biomass (D) from top 2010 salmon + habitat models. Plots include 1:1 lines (dashed) and regression lines (solid), as well as slope and intercept estimates and p-values. We show log-transformed data in panels A and C because predictive models were constructed using this data, and back transformed data in panels B and D because this is more illustrative of the actual effects of salmon on coastrange sculpin densities and biomass.
(PDF)

S4 Fig Scaled parameter estimates (circles) with 95% unconditional confidence intervals (lines) from the 95% confidence set of averaged multiple linear regression models of the effect of pink and chum salmon spawning density and stream habitat on coastrange and prickly sculpin densities (n = 81 and 60 respectively) and biomass (n = 85 and 87 respectively). Parameters are ordered by their relative importance (indicated on right) to the averaged model on a scale of zero.
(PDF)

S5 Fig Length-class frequency of prickly (black) and coastrange (grey) sculpin from 20 streams surveyed in summer 2010. (PDF)

S1 Table Results from AICc model competition between top models containing multi-year mean and single year salmon density terms in predicting numerical and biomass densities of coastrange sculpins. (DOCX)

S2 Table Variance inflation factor (VIF) and correlation coefficients for all explanatory variables included in sculpin population models. (DOCX)

S3 Table Stream name, coastrange and prickly sculpin numerical and biomass densities, pink + chum salmon density and bankfull width for streams included in this study. (DOCX)
