# Supplementary material for: Effects of Salmon-Derived Nutrients and Habitat Characteristics on Population Densities of Stream-Resident Sculpins
Source: PLoS One. 2015 Jun 1;10(6):e0116090. doi: 10.1371/journal.pone.0116090 (PMC4450874; doi:10.1371/journal.pone.0116090)
Supplement: S1 Fig — (PDF) [file pone.0116090.s001.pdf]

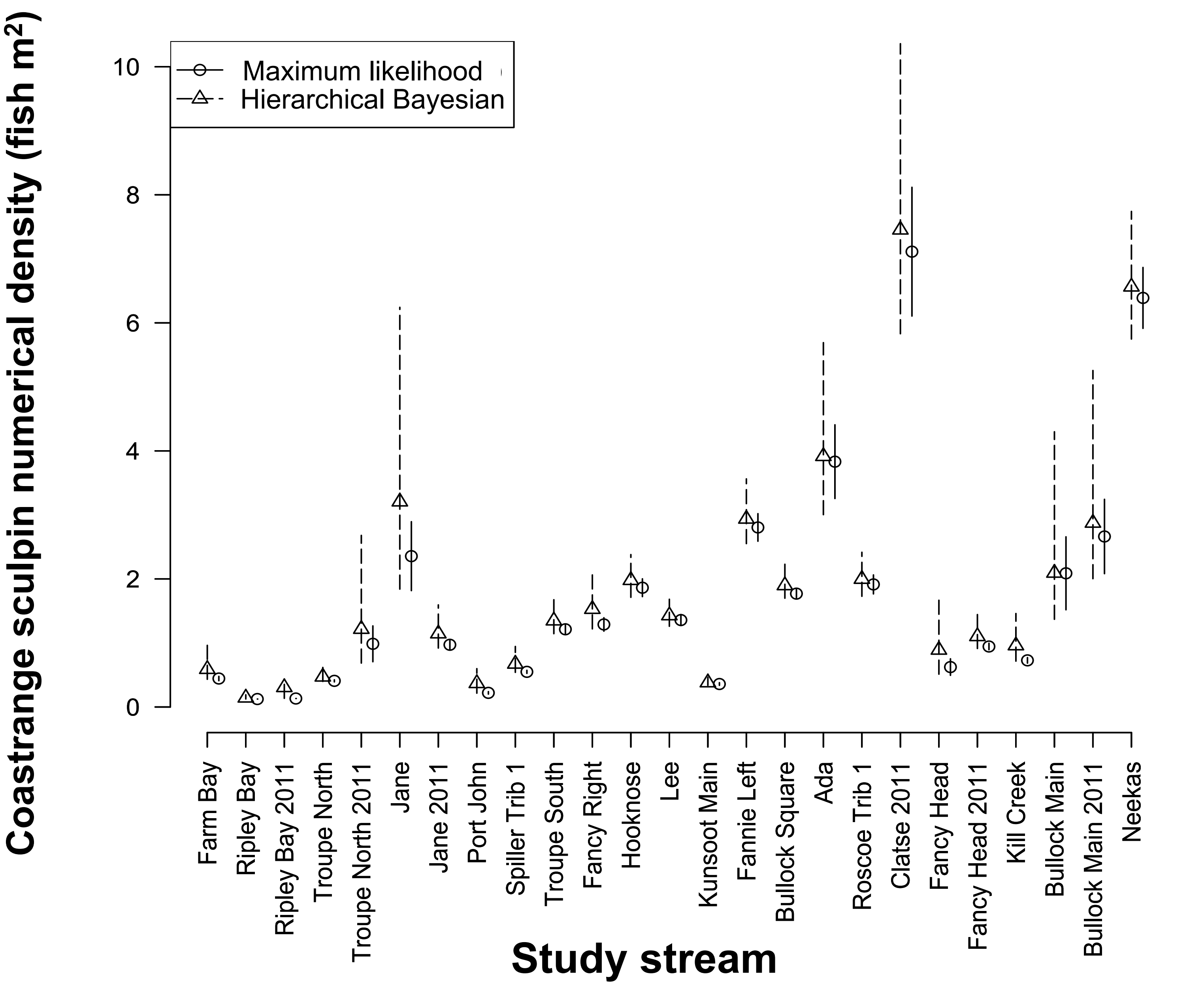

Figure S1. Coastrange sculpin density estimates and 95 % CI derived from maximum likelihood multiple-pass depletion methods (Carle and Strub 1978)(−o−) and estimates and 0.025 and 0.975 credible limits derived from Hierarchical Bayesian depletion models (Wyatt 2002)(---Δ---) for surveyed streams in 2010 and 2011.
