# Supplementary material for: Effects of Salmon-Derived Nutrients and Habitat Characteristics on Population Densities of Stream-Resident Sculpins
Source: PLoS One. 2015 Jun 1;10(6):e0116090. doi: 10.1371/journal.pone.0116090 (PMC4450874; doi:10.1371/journal.pone.0116090)
Supplement: S2 Fig — Dotted line is the 1:1 line and solid line is the regression line between the two methods. (PDF) [file pone.0116090.s002.pdf]

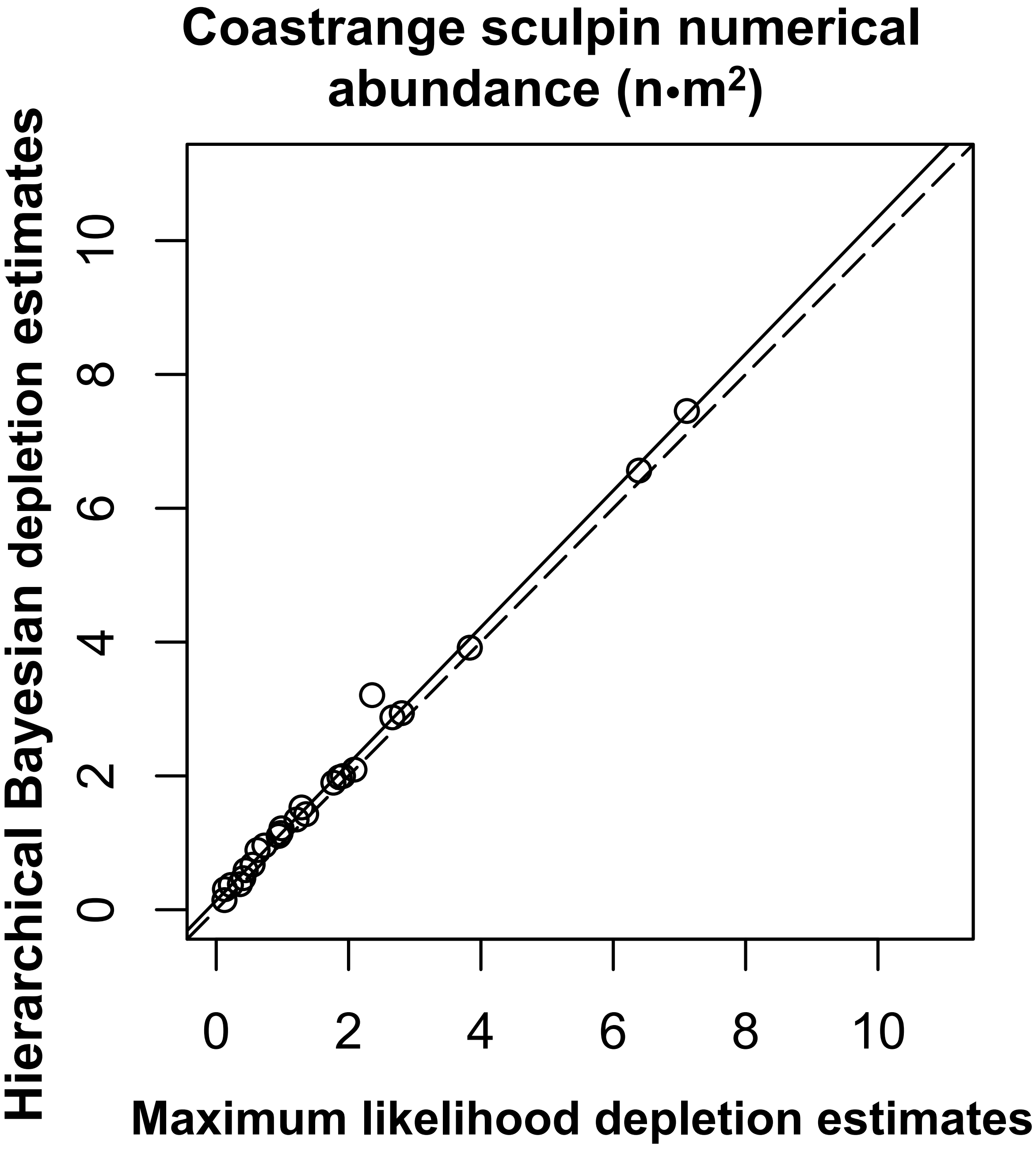

Figure S2. Coastrange sculpin density estimates derived from maximum likelihood multiple-pass depletion methods (Carle and Strub 1978) versus Hierarchical Bayesian depletion models (Wyatt 2002). Dotted line is the 1:1 line and solid line is the regression line between the two methods.
