# Supplementary material for: Effects of Salmon-Derived Nutrients and Habitat Characteristics on Population Densities of Stream-Resident Sculpins
Source: PLoS One. 2015 Jun 1;10(6):e0116090. doi: 10.1371/journal.pone.0116090 (PMC4450874; doi:10.1371/journal.pone.0116090)
Supplement: S3 Fig — Plots include 1:1 lines (dashed) and regression lines (solid), as well as slope and intercept estimates and p-values. We show log-transformed data in panels A and C because predictive models were constructed using this data, and back transformed data in panels B and D because this is more illustrative of the actual effects of salmon on coastrange sculpin densities and biomass. (PDF) [file pone.0116090.s003.pdf]

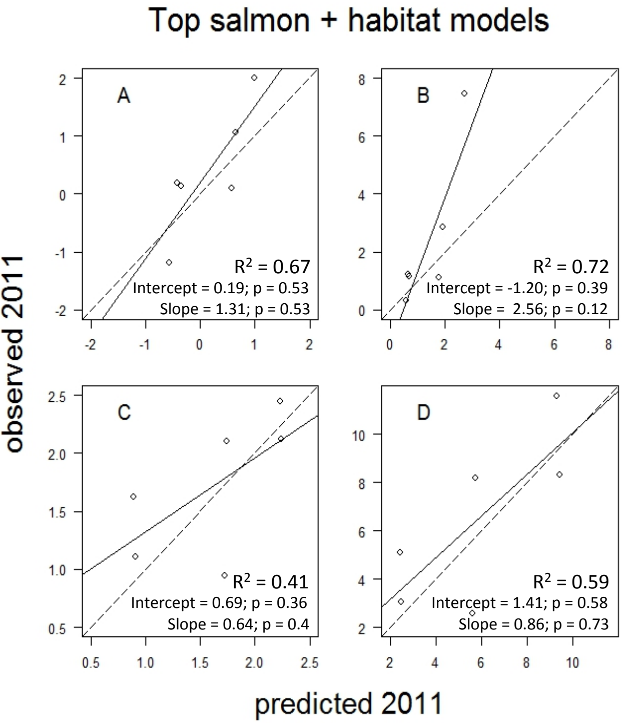

Figure S3. Observed 2011 versus predicted coastrange sculpin log (densities) (A), densities (B), log(biomass) (C), and biomass (D) from top 2010 salmon + habitat models.

Plots include 1:1 lines (dashed) and regression lines (solid), as well as slope and intercept estimates and p-values. We show log-transformed data in panels A and C because predictive models were constructed using this data, and back transformed data in panels B and D because this is more illustrative of the actual effects of salmon on coastrange sculpin densities and biomass.
