# Supplementary material for: Effects of Salmon-Derived Nutrients and Habitat Characteristics on Population Densities of Stream-Resident Sculpins
Source: PLoS One. 2015 Jun 1;10(6):e0116090. doi: 10.1371/journal.pone.0116090 (PMC4450874; doi:10.1371/journal.pone.0116090)
Supplement: S4 Fig — Parameters are ordered by their relative importance (indicated on right) to the averaged model on a scale of zero. (PDF) [file pone.0116090.s004.pdf]

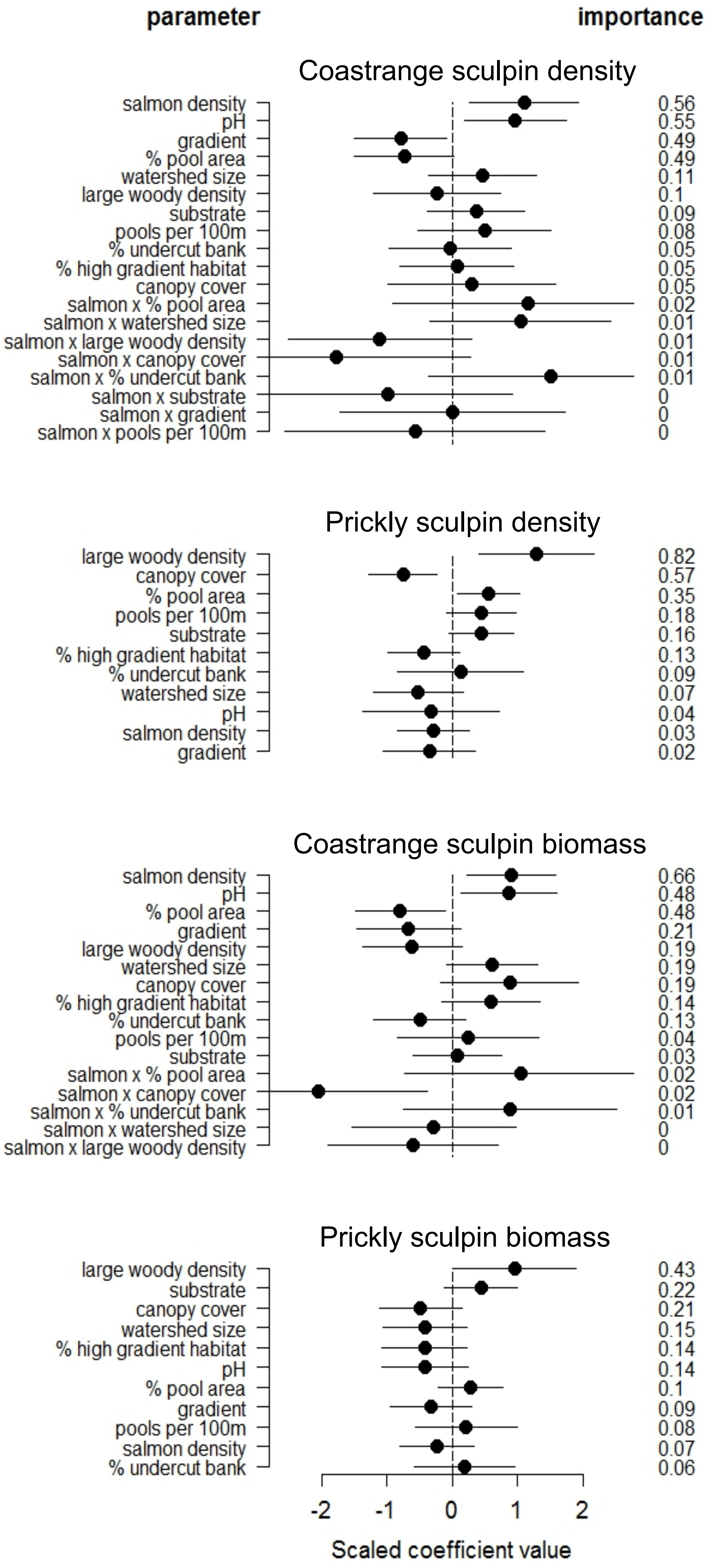

Figure S4. Scaled parameter estimates (circles) with 95% unconditional confidence intervals (lines) from the 95% confidence set of averaged multiple linear regression models of the effect of pink and chum salmon spawning density and stream habitat on coastrange and prickly sculpin densities (n = 81 and 60 respectively) and biomass (n = 85 and 87 respectively).

Parameters are ordered by their relative importance (indicated on right) to the averaged model on a scale of zero.
