# Supplementary material for: Effects of Salmon-Derived Nutrients and Habitat Characteristics on Population Densities of Stream-Resident Sculpins
Source: PLoS One. 2015 Jun 1;10(6):e0116090. doi: 10.1371/journal.pone.0116090 (PMC4450874; doi:10.1371/journal.pone.0116090)
Supplement: S5 Fig — (PDF) [file pone.0116090.s005.pdf]

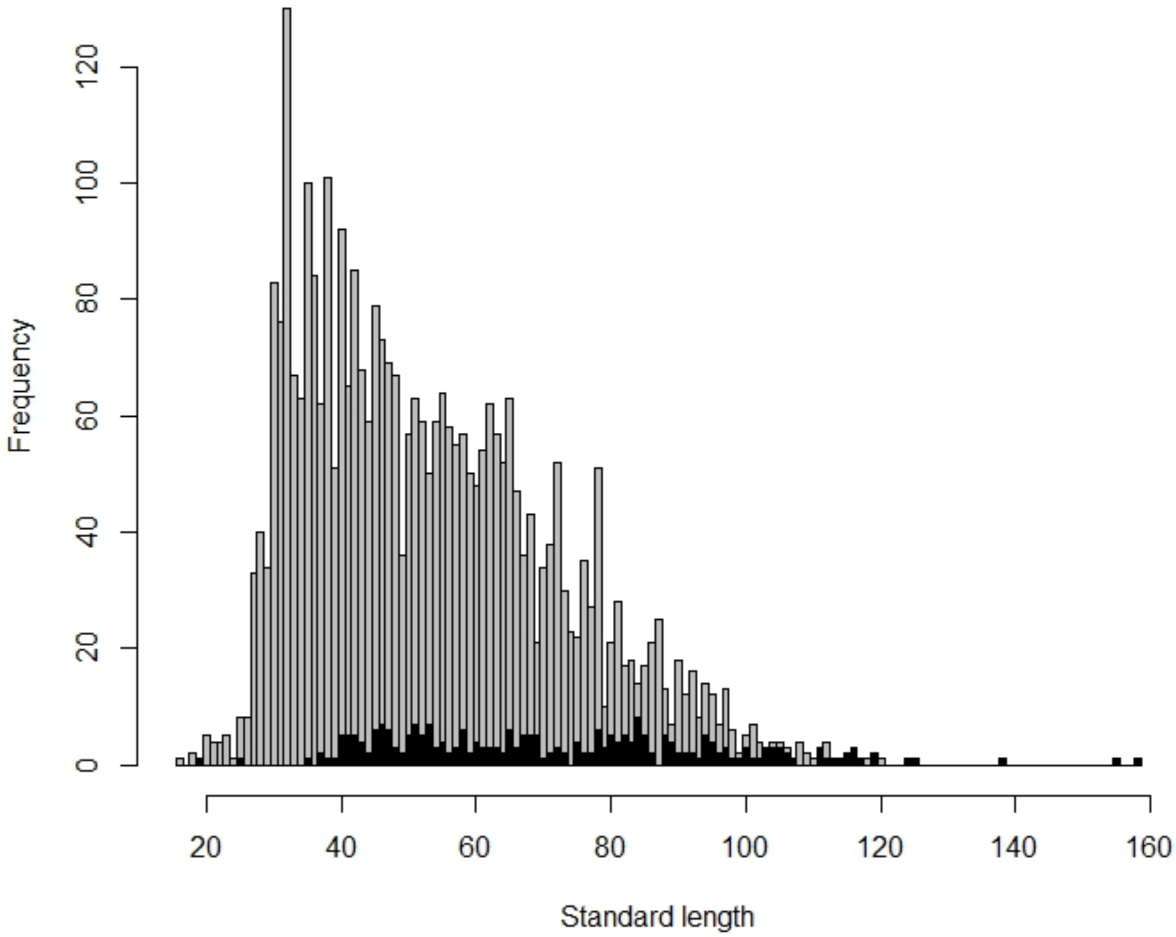

Figure S5. Length-class frequency of prickly (black) and coastrange (grey) sculpin from 20 streams surveyed in summer 2010.
