# Supplementary material for: Effects of Salmon-Derived Nutrients and Habitat Characteristics on Population Densities of Stream-Resident Sculpins
Source: PLoS One. 2015 Jun 1;10(6):e0116090. doi: 10.1371/journal.pone.0116090 (PMC4450874; doi:10.1371/journal.pone.0116090)
Supplement: S1 Table — (DOCX) [file pone.0116090.s006.docx]

**Table S1.** Results from AICc model competition between top models containing multi-year mean and single year salmon density terms in predicting numerical and biomass densities of coastrange sculpins.

| **Response** | **Model** | **K** | **Δ AICc** | **W_i_** | **R^2^** |
| --- | --- | --- | --- | --- | --- |
| **Coastrange sculpin density** | previous autumn salmon density + % pool area | 4 | 0.00 | 0.28 | 0.54 |
|  | 6 month decay 08-09 salmon density + % pool area | 4 | 0.56 | 0.21 | 0.53 |
|  | 12 month decay 08-09 salmon density + % pool area | 4 | 0.56 | 0.21 | 0.53 |
|  | 24 month decay 08-09 salmon density + % pool area | 4 | 0.72 | 0.20 | 0.53 |
|  | 5 year mean salmon density + % pool area | 4 | 2.30 | 0.09 | 0.48 |
| **Coastrange sculpin biomass** | 24 month decay 08-09 salmon density + % pool area + pH | 5 | 0.00 | 0.37 | 0.71 |
|  | previous autumn salmon density + % pool area + pH | 5 | 1.32 | 0.19 | 0.68 |
|  | 12 month decay 08-09 salmon density + % pool area + pH | 5 | 1.34 | 0.19 | 0.68 |
|  | 5 year mean salmon density + % pool area + pH | 5 | 1.53 | 0.17 | 0.68 |
|  | 6 month decay 08-09 salmon density + % pool area + pH | 5 | 3.13 | 0.08 | 0.65 |

Top model competition was not conducted for prickly sculpins because salmon density metrics were not included in high-ranking models.
