# Supplementary material for: Effects of Salmon-Derived Nutrients and Habitat Characteristics on Population Densities of Stream-Resident Sculpins
Source: PLoS One. 2015 Jun 1;10(6):e0116090. doi: 10.1371/journal.pone.0116090 (PMC4450874; doi:10.1371/journal.pone.0116090)
Supplement: S2 Table — (DOCX) [file pone.0116090.s007.docx]

**Table S2.** Variance inflation factor (VIF) and correlation coefficients for all explanatory variables included in sculpin population models.

| **Variable^1^** | **VIF** | **Prev-**  **ious**  **autumn salmon density** | **5 yr mean salmon density** | **Water-shed size PC1** | **Sub-**  **strate PC1** | **Pools per 100m** | **% pool area** | **% under-**  **cut bank** | **% high grad**  **-ient habitat** | **Mean grad-**  **ient degrees** | **Large wood**  **density** | **Canopy cover** | **pH** |
| --- | --- | --- | --- | --- | --- | --- | --- | --- | --- | --- | --- | --- | --- |
| **Previous autumn**  **salmon density** | *21.64* | 1.00 | 0.96 | 0.27 | -0.15 | -0.23 | 0.00 | 0.00 | -0.27 | -0.30 | 0.10 | 0.05 | 0.44 |
| **5 yr mean**  **salmon density** | *21.56* | 0.96 | 1.00 | 0.22 | -0.15 | -0.29 | 0.00 | -0.02 | -0.20 | -0.31 | 0.17 | 0.04 | 0.46 |
| **Watershed**  **size PC** | *3.73* | 0.27 | 0.22 | 1.00 | 0.03 | -0.52 | -0.48 | -0.32 | 0.17 | -0.25 | -0.46 | 0.31 | 0.33 |
| **Substrate PC1** | *3.17* | -0.15 | -0.15 | 0.03 | 1.00 | -0.32 | 0.06 | -0.37 | 0.30 | 0.02 | -0.38 | 0.27 | -0.11 |
| **Pools per 100m** | *2.68* | -0.23 | -0.29 | -0.52 | -0.32 | 1.00 | 0.14 | 0.36 | 0.02 | 0.34 | 0.25 | -0.46 | -0.40 |
| **% pool area** | *3.07* | 0.00 | 0.00 | -0.48 | 0.06 | 0.14 | 1.00 | 0.44 | -0.39 | -0.12 | 0.53 | -0.01 | -0.13 |
| **% undercut**  **bank** | *2.78* | 0.00 | -0.02 | -0.32 | -0.37 | 0.36 | 0.44 | 1.00 | -0.49 | -0.15 | 0.30 | -0.36 | -0.46 |
| **% high**  **gradient habitat** | *4.91* | -0.27 | -0.20 | 0.17 | 0.30 | 0.02 | -0.39 | -0.49 | 1.00 | 0.57 | -0.03 | -0.06 | 0.11 |
| **Mean gradient**  **degrees** | *2.96* | -0.3 | -0.31 | -0.25 | 0.02 | 0.34 | -0.12 | -0.15 | 0.57 | 1.00 | 0.28 | 0.01 | 0.10 |
| **Large wood density** | *4.01* | 0.1 | 0.17 | -0.46 | -0.38 | 0.25 | 0.53 | 0.3 | -0.03 | 0.28 | 1.00 | -0.06 | 0.23 |
| **Canopy cover** | *2.27* | 0.05 | 0.04 | 0.31 | 0.27 | -0.46 | -0.01 | -0.36 | -0.06 | 0.01 | -0.06 | 1.00 | 0.49 |
| **pH** | *3.23* | 0.44 | 0.46 | 0.33 | -0.11 | -0.4 | -0.13 | -0.46 | 0.11 | 0.10 | 0.23 | 0.49 | 1.00 |

*^1^High VIF scores for salmon metrics resulted from high correlation among them, but did not affect models as only previous autumn salmon density was used in our main analyses.*
