# Supplementary material for: Effects of Salmon-Derived Nutrients and Habitat Characteristics on Population Densities of Stream-Resident Sculpins
Source: PLoS One. 2015 Jun 1;10(6):e0116090. doi: 10.1371/journal.pone.0116090 (PMC4450874; doi:10.1371/journal.pone.0116090)
Supplement: S3 Table — (DOCX) [file pone.0116090.s008.docx]

**Table S3.** Stream name, coastrange and prickly sculpin numerical and biomass densities, pink + chum salmon density and bankfull width for streams included in this study.

| **Stream** | **Coastrange sculpin^1^** | | **Prickly sculpin^2^** | | **Pink + Chum salmon** | **Bankfull width (m)** |
| --- | --- | --- | --- | --- | --- | --- |
|  | **Density**  **(#**•**m^-2^)** | **Biomass**  **(g**•**m^-2^)** | **Density**  **(#**•**m^-2^)** | **Biomass**  **(g**•**m^-2^)** | **Previous autumn**  **density**  **(kg**•**m^-2^)** |  |
| **2010** |  |  |  |  |  |  |
| Ada | 3.92 | 11.88 | 0.19 | 1.72 | 1.72 | 11.1 |
| Bullock Main | 2.09 | 10.40 | 0.17 | 1.42 | 3.05 | 10.9 |
| Bullock Square | 1.90 | 8.32 | − | − | 1.12 | 8.4 |
| Codville | − | − | 0.62 | 3.20 | 0.17 | 3.3 |
| Fancy Head | 0.89 | 6.04 | 0.25 | 3.05 | 1.15 | 5.5 |
| Fancy Right | 1.53 | 4.22 | 0.14 | 1.17 | 0.95 | 4.8 |
| Fannie Left | 2.94 | 5.48 | 0.14 | 1.45 | 1.19 | 12.8 |
| Farm Bay | 0.59 | 2.17 | − | − | 0.00 | 6.4 |
| Hooknose | 1.98 | 8.04 | 0.18 | 3.13 | 0.49 | 16.9 |
| Jane | 3.21 | 6.15 | 0.41 | 2.90 | 0.02 | 4.6 |
| Kill Creek | 0.96 | 3.26 | − | − | 2.18 | 3.5 |
| Lee | 1.43 | 7.84 | − | − | 0.67 | 12.4 |
| Neekas | 6.56 | 11.52 | 0.26 | 2.57 | 8.06 | 17.7 |
| Port John | 0.37 | 0.90 | − | − | 0.00 | 3.3 |
| Quartcha | 2.33 | 16.05 | − | − | 0.11 | 21.7 |
| Ripley Bay | 0.14 | 1.45 | 0.15 | 1.65 | 0.00 | 14.7 |
| Roscoe Trib 1 | 2.00 | 16.22 | − | − | 1.25 | 14.1 |
| Spiller Trib 1 | 0.67 | 3.09 | − | − | 0.13 | 7.7 |
| Troupe North | 0.47 | 1.55 | − | − | 0.01 | 4.4 |
| Troupe South | 1.35 | 6.31 | 0.17 | 2.27 | 0.35 | 4.1 |
| **2011** |  |  |  |  |  |  |
| Bullock Main | 2.87 | 8.32 | 0.10 | 0.19 | 0.96 | 10.9 |
| Clatse | 7.45 | 11.56 | 0.05 | 0.39 | 1.43 | 22.8 |
| Fancy Head | 1.10 | 8.17 | − | − | 0.78 | 5.5 |
| Jane | 1.15 | 2.56 | − | − | 0.003 | 4.6 |
| Ripley Bay | 0.31 | 3.03 | − | − | 0 | 14.7 |
| Troupe North | 1.22 | 5.07 | − | − | 0 | 4.4 |

^1^*Coastrange sculpins were caught in 21 streams but population estimates were only possible from 20 of them*

*^2^Prickly sculpin were caught in 14 streams but population estimates were only possible in 12 of them*
